# Transcriptome Analysis of Immune Receptor Activation and Energy Metabolism Reduction as the Underlying Mechanisms in Interleukin-6-Induced Skeletal Muscle Atrophy

**DOI:** 10.3389/fimmu.2021.730070

**Published:** 2021-09-06

**Authors:** Hualin Sun, Junjie Sun, Ming Li, Lei Qian, Lilei Zhang, Ziwei Huang, Yuntian Shen, Betty Yuen-Kwan Law, Liang Liu, Xiaosong Gu

**Affiliations:** ^1^State Key Laboratory of Quality Research in Chinese Medicine, Macau University of Science and Technology, Macau, Macau, SAR China; ^2^Key Laboratory of Neuroregeneration of Jiangsu and Ministry of Education, Co-Innovation Center of Neuroregeneration, Nantong University, National Medical Products Administration Key Laboratory for Research and Evaluation of Tissue Engineering Technology Products, Jiangsu Clinical Medicine Center of Tissue Engineering and Nerve Injury Repair, Nantong, China; ^3^Department of Laboratory Medicine, Binhai County People’s Hospital Affiliated to Kangda College of Nanjing Medical University, Yancheng, China; ^4^Division of Sports Medicine and Adult Reconstructive Surgery, Department of Emergency, Department of Orthopedic Surgery, Nanjing Drum Tower Hospital, The Affiliated Hospital of Nanjing University Medical School, Nanjing, China

**Keywords:** interleukin-6, muscle atrophy, transcription factor, energy metabolism, inflammation

## Abstract

**Background:**

Inflammation may trigger skeletal muscle atrophy induced by cancer cachexia. As a pro-inflammatory factor, interleukin-6 may cause skeletal muscle atrophy, but the underlying molecular mechanisms have not been explored.

**Methods:**

In this experimental study, we used adult male ICR mice, weighing 25 ± 2 g, and the continuous infusion of interleukin-6 into the tibialis anterior muscle to construct a skeletal muscle atrophy model (experimental group). A control group received a saline infusion. RNA-sequencing was used to analyze the differentially expressed genes in tissue samples after one and three days. Gene Ontology and the Kyoto Encyclopedia of Genes and Genomes analysis were applied to define the function of these genes, and protein-protein interaction analysis was performed to identify potential transcription factors. Fluorescence microscopy was used to determine the muscle fiber cross-sectional area after 14 days.

**Results:**

Continuous infusion of interleukin-6 for 14 days caused significant muscle atrophy. RNA-sequencing found 359 differentially expressed genes in the 1- and 3-day tissue samples and 1748 differentially expressed genes only in the 3-day samples. Functional analysis showed that the differentially expressed genes found in both the 1- and 3-day samples were associated with immune receptor activation, whereas the differentially expressed genes found only in the 3-day sample were associated with reduced energy metabolism. The expression of multiple genes in the oxidative phosphorylation and tricarboxylic acid cycle pathways was down-regulated. Furthermore, differentially expressed transcription factors were identified, and their interaction with interleukin-6 and the differentially expressed genes was predicted, which indicated that STAT3, NF-κB, TP53 and MyoG may play an important role in the process of interleukin-6-induced muscle atrophy.

**Conclusions:**

This study found that interleukin-6 caused skeletal muscle atrophy through immune receptor activation and a reduction of the energy metabolism. Several transcription factors downstream of IL-6 have the potential to become new regulators of skeletal muscle atrophy. This study not only enriches the molecular regulation mechanism of muscle atrophy, but also provides a potential target for targeted therapy of muscle atrophy.

## Background

Skeletal muscle homeostasis refers to the balance between anabolism and catabolism in response to endogenous and exogenous stimuli and enables maintaining movement ability in mammals (1, 2). Skeletal muscles have strong plasticity, and consistent physical exercise can increase muscle mass and muscle strength. However, the balance can be disturbed by various factors, such as cancer cachexia, mechanical damage, denervation, bed rest, and diabetes that trigger skeletal muscle atrophy (3). Cancer cachexia is a metabolic disease characterized by systemic inflammation and skeletal muscle atrophy, which seriously affects patients’ quality of life and is related to their survival rate (4). Cachexia is very common in patients with lung tumors, especially in those with small cell lung cancer. Lung cancer patients with cachexia who develop skeletal muscle atrophy have a shorter overall survival rate and less favorable response to PD-1 antibody treatment (5). Alleviating skeletal muscle atrophy may slow down the progression of cancer cachexia. However, there are no drugs that effectively treat skeletal muscle atrophy to date, and in-depth studies of the molecular mechanisms underlying skeletal muscle atrophy are warranted to facilitate the development of specific therapeutic approaches.

Skeletal muscle atrophy is a complex process involving myriads of molecules. Generally, skeletal muscle atrophy is induced by the activation of different protein degradation pathways, including the ubiquitin-proteasome pathway, autophagy-lysosome pathway, and calpain pathway (6, 7). We previously analyzed the molecular changes in denervation-induced muscle atrophy using microarray testing, and found that the muscle atrophy process can be divided into four stages, namely oxidative stress stage (<12 hours), inflammation stage (24 hours), atrophy stage (3–7 days), and atrophic fibrosis stage (7days–) (8). A study using RNA-sequencing found that atrophy can be observed within 36 hours to 3 days after denervation (9). The authors concluded that the massive inflammatory response about 24 hours after denervation might trigger skeletal muscle atrophy. In a denervation-induced muscle atrophy model, Lewis lung carcinoma cell-derived extracellular vesicles caused C2C12 myotube atrophy in a dose-dependent manner (10). Extracellular vesicles directly release interleukin (IL)-6 into the C2C12 myotubes and then activate the signal transducer and activator of the transcription 3 (STAT3) signaling pathway, leading to atrophy. These findings imply that inflammation plays an important role in inducing skeletal muscle atrophy.

IL-6 is a common pro-inflammatory factor that is normally secreted by T cells, macrophages, fibroblasts, and endothelial cells and remains stable in tissues and blood. However, it may quickly accumulate locally in acute inflammation reactions, and its expression level may increase 100-fold and even 1000-fold. The serum levels and muscle tissue levels of IL-1β, tumor necrosis factor-α (TNF-α), and especially IL-6 are significantly increased in patients with cancer cachexia, which aggravates the condition and negatively affects patients’ prognosis (11). IL-6 is a signal molecule: In the classic IL-6 signaling pathway, extracellular IL-6 binds to the IL-6 receptor (IL-6R; CD126) on the cell membrane, activating it to recruit gp130 and forming dimers that transmit intracellular signals (12). The Janus-activated kinase/signal transducer and activator of transcription (JAK/STAT) pathway is recognized as a downstream pathway of IL-6 signaling and can change the expression of a large number of genes (13).

Numerous studies have focused on the effects of IL-6 on skeletal muscle physiology (14–17). Our previous studies have demonstrated that denervation can induce a substantial accumulation of IL-6 in the concerned skeletal muscles, and local injection of IL-6 into the skeletal muscle of mice induced atrophy (18). Moreover, the upregulation of IL-6 in a variety of patients with muscle atrophy and mammalian atrophy models suggests that IL-6 plays a key role in skeletal muscle atrophy (19). However, few studies have focused on the underlying mechanisms of IL-6-induced skeletal muscle atrophy.

In this study, we aimed to examine the underlying molecular mechanisms of IL-6-induced skeletal muscle atrophy in a mouse model. Therefore, the continuous infusion of interleukin-6 or saline into the tibialis anterior muscle was used to construct a skeletal muscle atrophy model. RNA-sequencing was used to analyze the differentially expressed genes in tissue samples after one and three days. Bioinformatics analysis revealed key IL-6 downstream pathways and identified potential transcription factors. This study not only further enhances our understanding of the molecular regulation mechanisms of muscle atrophy but provides new potential regulators of this process.

## Methods

### Ethical Statement

All the experiments involving mice in this study were performed under a project license (No. S20200312-003) granted by the Animal Ethics Board of Nantong University, in compliance with national guidelines for the care and use of animals.

### Animals and Experiment

Adult male ICR mice, weighing 25 ± 2 g, were provided by the Laboratory Animal Center of Nantong University, Nantong, China. The animals were reared in a specific pathogen-free animal house, kept at 23 ± 2°C and 60% air humidity, and a 12-hour light/dark cycle with free access to standard rodent chow and water. A total of 15 mice were randomly divided into two groups. 6 mice in the first group were injected with PBS as control, and 9 mice in the second group were injected with rIL-6. Samples were taken on 1 day, 3 days and 14 days respectively for RNAseq or morphological analysis (n = 3).

Under anesthesia, the left tibialis anterior (TA) muscle was exposed in all mice. An osmotic pump (ALZET^®^ osmotic pump, model 2002, DURECT Corporation, Cupertino, CA, USA) was implanted subcutaneously in all mice. In the experimental group, recombinant murine IL-6 (rIL-6) (Beyotime Biotech Inc., Haimen, China) was continuously infused into the left TA muscle through a catheter (inside diameter 0.006 inch, DURECT Corporation, Cupertino, CA, USA) at a flow rate of 4.5 pg/h as described in previous publications (20, 21). In the control group, phosphate-buffered saline was used instead at the same infusion rate. After one, three, and fourteen days, TA muscle tissue samples were harvested in all mice under anesthesia, weighed, snap-frozen in liquid nitrogen, and stored at −80°C.

### Determination of Muscle Fiber Cross-Sectional Area

The 14-day muscle tissue samples were used for morphological analysis. Laminin staining was performed to determine the muscle fiber cross-sectional area (CSA), as previously described (18, 22). Briefly, the TA muscle samples were obtained, fixed, and cut into 10-μm thick cryo-sections. Next, the sections were incubated with an anti-laminin antibody (Abcam plc., Cambridge, UK) at 4°C for 12 hours and afterward with a fluorescent secondary antibody (Invitrogen Alexa Fluor, Thermo-Fisher Scientific, Waltham, MA, USA) at room temperature for one hour. The sections were investigated and photographed under fluorescence microscopy (Zeiss Microscopy, Jena, Germany), and the muscle fiber CSA was determined using ImageJ software (National Institutes of Health, Bethesda, MD, USA).

### RNA Extraction and RNA-seq

We used the samples harvested after one and three days for transcriptome sequencing. The TRIzol method was used for total RNA extraction. The frozen muscle tissue was immersed with TRIzol™ Reagent (Thermo-Fisher Scientific, Waltham, MA, USA) and was crushed using a tissue crusher until there were no visible solids to ensure that the muscle tissue was completely dissolved. The RNA was extracted with chloroform and isopropanol in sequence, eluted with 75% ethanol, and finally dissolved in diethylpyrocarbonate water. We used 2 μg total RNA to enrich mature mRNA using magnetic beads carrying Oligo (dT), and the enriched RNA was interrupted after purification. Random primer fragments were used as a template to synthesize the first-strand cDNA, which was then used as a template to synthesize double-stranded DNA. A series of treatments such as end repair, A-tailing, and connection of sequencing adapters were performed, and the samples were amplified using polymerase chain reaction to complete library construction. Quality testing was conducted using the Agilent 2100 Bioanalyzer (Agilent Technologies, Inc., Santa Clara, CA, USA). The Illumina HiSeq X Ten sequencer (Illumina Inc., San Diego, CA, USA) was used for sequencing, and 150 bp paired-end data was obtained. The gene expression dataset is available on ArrayExpress and the accession number is E-MTAB-10794.

### Preliminary Data Processing

For the sequencing data output of the Illumina platform, the Trimmomatic software (23) was used to preprocess raw data and eliminate the influence of data errors, including removing reads containing adapters, low-quality reads, and low-quality bases at the 3’ and 5’ ends. HISAT2 (24) was used to compare clean reads with the designated reference genome to obtain information on the reference genome or gene position and feature information on unique sequences in the sequence sample.

### Identification of Differentially Expressed Genes

The expression level of protein-coding genes was estimated by counting the sequences located in the exon region of the protein-coding gene. We used HTSeq-count software (25) to count reads mapped to protein-coding genes in each sample and the Cufflinks suite of software (26) to calculate the protein-coding gene expression value of fragments per kb per million reads. To identify the differentially expressed genes (DEGs), the DESeq software (27) was used to normalize the counts of reads in each sample gene and calculate the fold change first, before the negative binomial distribution test was used to calculate the P-value for the difference in the number of reads. In this study, a significant difference in the DEGs and transcription factors was defined as P < 0.05 and fold change > 2.

### Functional Analysis

VENNY (http://bioinfogp.cnb.csic.es/tools/venny/index.html) was used to filter the genes of interest in the DEGs that had been defined. We used the DAVID Bioinformatics Resources 6.8 tool (28) to analyze the function of the gene set and ran different analyses (biological process (BP), cell component (CC), molecular function, and Kyoto Encyclopedia of Genes and Genomes (KEGG)) to identify the pathways associated with the DEGs. Gene Ontology (GO) analysis was performed to analyze the up-regulated and down-regulated genes. The P-value corresponding to each pathway was employed to define the relevance between the gene set and the pathway.

### Interaction Analysis of Transcription Factors

The protein-protein interaction file was downloaded from the String database (https://www.string-db.org/), and each pair containing a transcription factor target and a molecule target was extracted. The score was calculated as previously described (29). A higher score indicated a stronger interaction, with a maximum value of 1000. Relationship pairs containing a single molecule and a target molecule were collected in one file, and Cytoscape software (30) was used to draw the interaction diagram.

### Statistical Analysis

The statistical analysis was performed using SPSS Statistics for Windows, version 17.0 (SPSS Inc., Chicago, IL, USA). T-test was used to calculate the P-values for the CSA differences between the groups. For intergroup comparison, a P < 0.05 was considered statistically significant. Data in the histograms were expressed as the mean ± standard error of mean (SEM).

## Results

### Identify DEGs Induced By IL-6 in Skeletal Muscle

**Figure 1:**
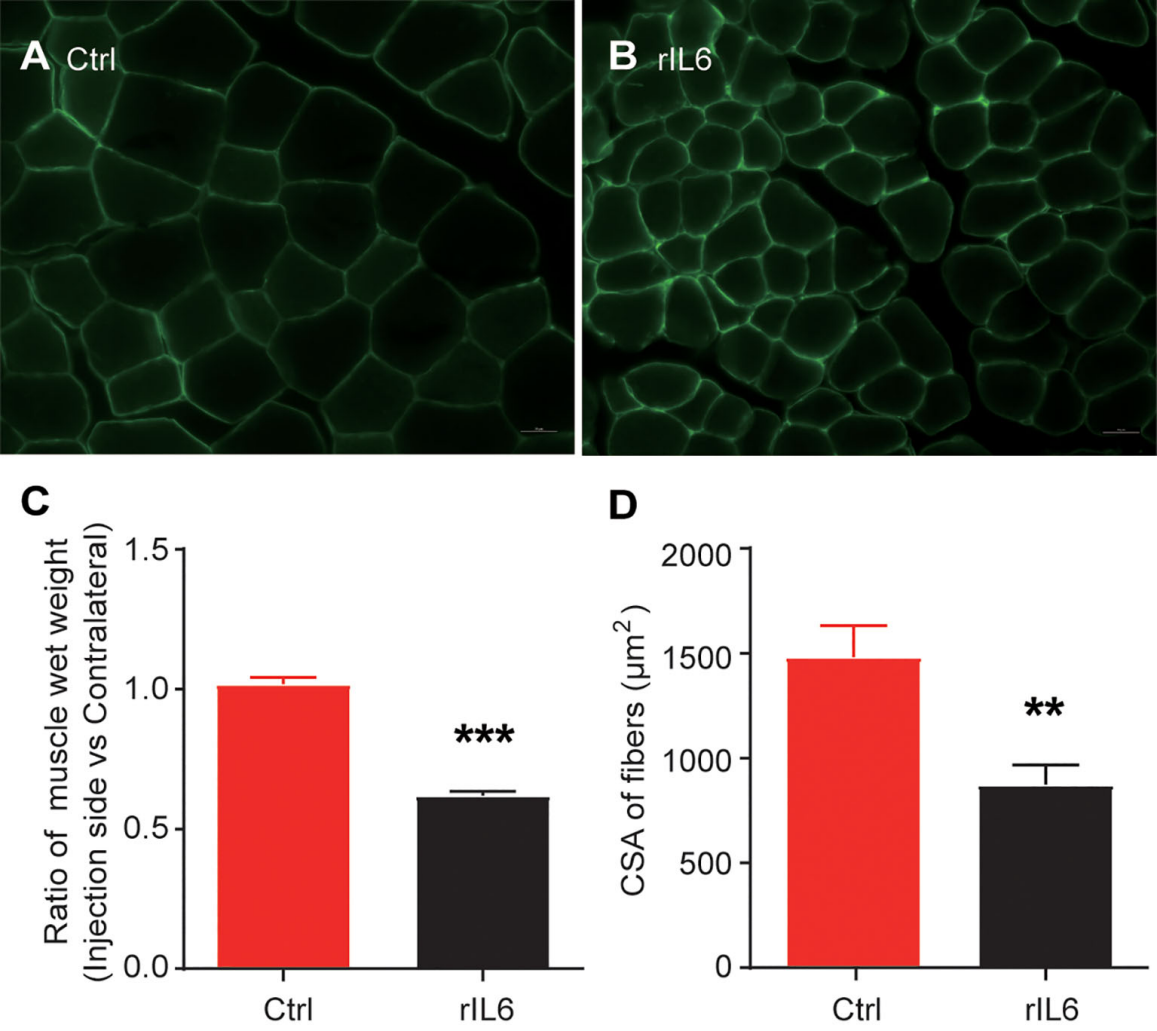
Interleukin-6 (IL-6) induces skeletal muscle atrophy. **(A)** Laminin immunohistochemical staining of the tibialis anterior muscle of mice in the control group (injected with phosphate-buffered saline); scale bars = 20 μm. **(B)** Laminin immunohistochemical staining of the tibialis anterior muscle. **(C)** Results of the skeletal muscle wet-weight ratio between the experimental group and the control group. **(D)** Results of the comparison of the cross-sectional muscle fiber area between the experimental group and the control group. Statistical results, the bar graph represents the mean ± standard error of the mean, T-test was used to calculate the P-values, n = 3, **P < 0.01, ***P < 0.001, experimental group *vs.* control group. CSA, cross-sectional area; Ctrl, control; IL-6, interleukin-6; rIL-6, recombinant interleukin-6.

**Figure 2:**
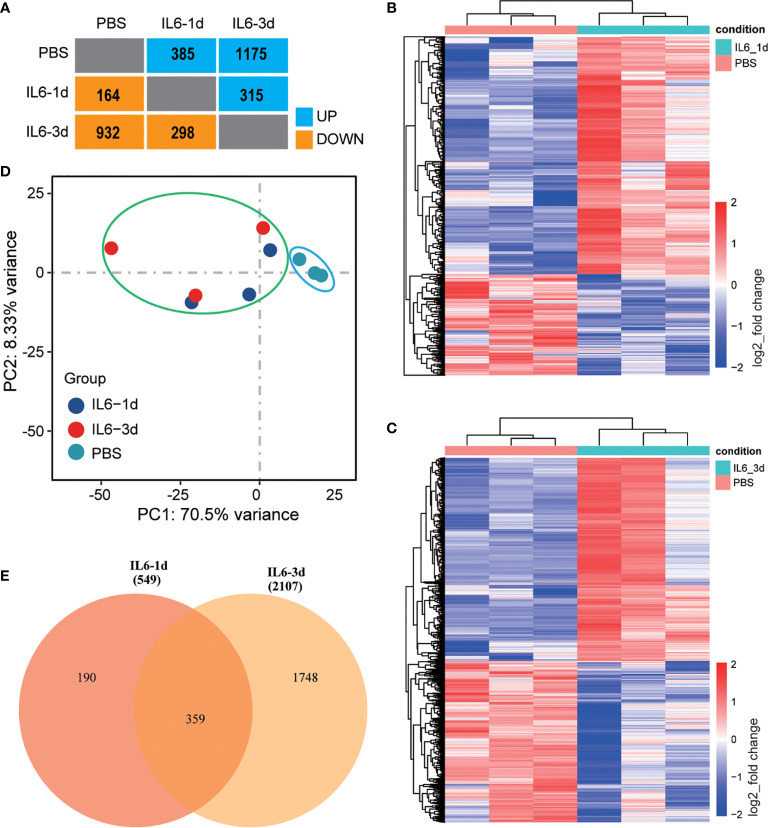
Identification of differentially expressed genes (DEGs) in skeletal muscle after interleukin-6 (IL-6) infusion. **(A)** A summary of the number of DEGs after one and three days of IL-6 infusion in the intervention group compared with the control group. Blue indicates up-regulated genes, and orange indicates down-regulated genes. **(B, C)** Heat maps of all differentially expressed genes after one day **(B)** and three days **(C)** of IL-6 infusion. Red indicates up-regulated expression, and blue indicates down-regulated expression. **(D)** Principal component analysis of a single-sequencing sample. The shorter the sample clustering distance, the more similar the samples. The control group is marked by a blue ellipse and the experimental group by a green ellipse. **(E)** VENNY diagram of the overlap of DEGs after one and three days of IL-6 infusion. IL-6, interleukin-6; PBS, phosphate-buffered saline.

After 14 days, significant muscle atrophy was observed in the experimental group. Compared with the control group, the cross-sectional area of ​​muscle fibers was reduced by 41% (P < 0.01), and the wet-weight ratio was reduced by 38% (P < 0.001) (**Figure 1**). The bioinformatics analysis identified 385 up-regulated and 164 down-regulated DEGs in the 1-day muscle samples and 1175 up-regulated and 932 down-regulated DEGs in the 3-day samples. Compared with the 1-day sample, there were 315 up-regulated and 298 down-regulated different genes in the 3-day sample (**Figure 2A**). The heatmap results of these DEGs are shown in **Figures 2B, C**. We also performed a principal component analysis (PCA) on these samples. The PCA results showed that the experimental group could be clearly separated from the control group, implying good intragroup consistency and significant intergroup diversity (**Figure 2D**). The DEGs to be studied in more detail were divided into three categories: class I included 190 DEGs after one day of IL-6 infusion, class II included the 359 DEGs found both after one and three days of IL-6 infusion, and class III included 1748 DEGs after three days of IL-6 infusion (**Figure 2E**).

### Permanently Changed Genes and Their Biological Functions When IL-6 Was Infused for One and Three Days

**Figure 3:**
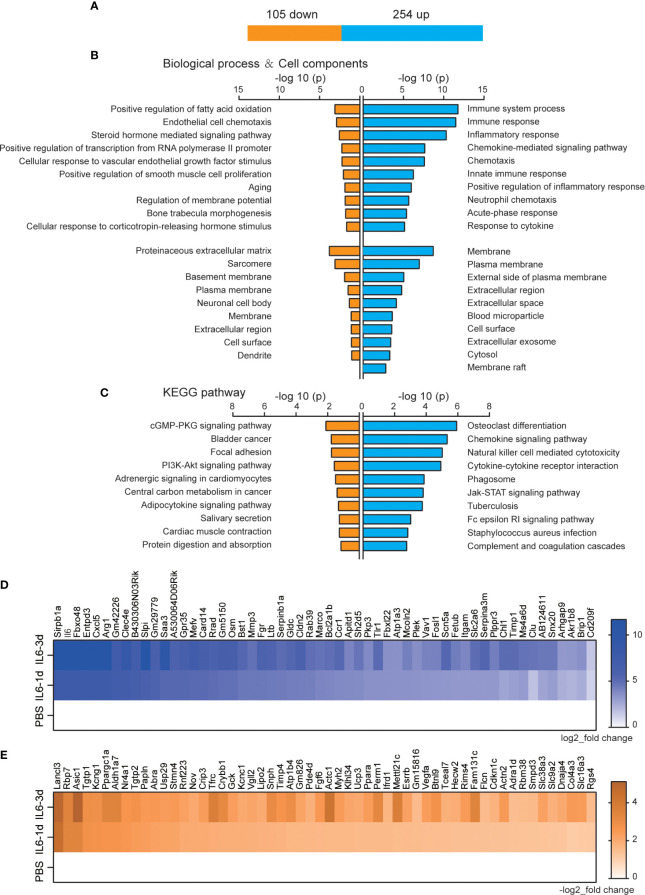
Permanently changed genes and their biological functions after one and three days of interleukin-6 (IL-6) infusion. **(A)** The number of genes that were changed after both one and three days of infusion. Blue indicates up-regulated genes, and orange indicates down-regulated genes. **(B)** The biological process of up-regulated and down-regulated differentially expressed genes (DEGs) and the Gene Ontology (GO) analysis results of the cell components. Either the top 10 or all significant GO terms are displayed. **(C)** The Kyoto Encyclopedia of Genes and Genomes pathway analysis results of the up-regulated and down-regulated DEGs. The top 10 GO terms are displayed. **(D)** Heat map of up-regulated DEGs with an increase in upregulation folds. **(E)** Heat map of down-regulated DEGs with an increase in downregulation folds. For **(D, E)**, data were normalized to PBS controls. KEGG, Kyoto Encyclopedia of Genes and Genomes; PBS, phosphate-buffered saline; down, down-regulated; up, up-regulated.

There were 359 DEGs that were observed after both one and three days of IL-6 infusion, including 254 up-regulated and 105 down-regulated genes (**Figure 3A**). The GO analysis of the up-regulated and down-regulated genes in the categories of BP and CC used P-values to reflect the differences between these pathways and the DEGs. All P-values were concentrated in a column chart (**Figures 3B, C**). The down-regulated genes could not be functionally aggregated, and the up-regulated genes were related to multiple immunology pathways, such as immune system process, immune response, inflammatory response, and chemokine-mediated signaling pathways (**Figure 3B**). In the CC analysis, these DEGs were mainly distributed on the cell membrane, suggesting that IL-6 activates multiple immune receptors (**Figure 3B**, **Supplementary File 1**). The down-regulated DEGs could not be functionally aggregated. The up-regulated DEGs were mainly related to osteoclast differentiation, chemokine signaling pathways, natural killer cell-mediated cytotoxicity, cytokine-cytokine receptor interactions, phagosomes, the JAK-STAT signaling pathway, tuberculosis, the Fc epsilon RI signaling pathway, *Staphylococcus aureus* infection, and complement and coagulation cascade pathways (**Figure 3C**). The JAK-STAT signaling pathway has been confirmed to cause a series of atrophic phenotypes in the skeletal muscle due to IL-6 activation (20, 31). A variety of inflammatory response pathways, especially the expression of receptors, were activated after both one and three days of IL-6 infusion. Since IL-6 was infused continuously, these changed genes were more likely to be targets directly affected by IL-6. We used heatmaps to enumerate all DEGs with a higher multiple of gene differences after three days than after one day (**Figures 3D, E**).

### Biological Function of the DEGs Newly Discovered After Three Days of IL-6 Infusion

**Figure 4:**
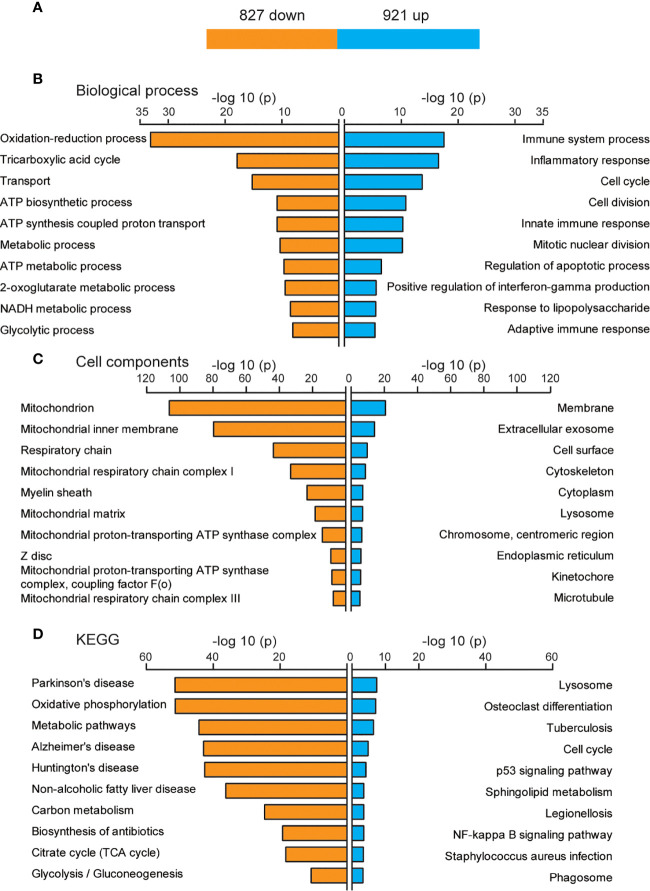
Biological functions of newly discovered differentially expressed genes (DEGs) after three days of interleukin-6 (IL-6) infusion. **(A)** The number of DEGs that were newly discovered after three days of IL-6 infusion. Blue indicates up-regulated genes, and orange indicates down-regulated genes. **(B)** The GO analysis results of up-regulated and down-regulated DEGs according to the biological process. **(C)** The GO analysis results of the up-regulated and down-regulated DEGs according to the cell component. **(D)** Kyoto Encyclopedia of Genes and Genomes (KEGG) pathway analysis of the up-regulated and down-regulated DEGs. The top 10 GO or KEGG terms are displayed. ATP, adenosine triphosphate; up, up-regulated.

The function of DEGs that only appeared after three days of IL-6 infusion, including 921 up-regulated and 827 down-regulated genes, is shown in **Figure 4A**. The GO analysis results of the BP category showed that up-regulated genes were mainly related to immune system processes, and down-regulated genes were related to energy metabolism processes, such as oxidation-reduction processes, the tricarboxylic acid cycle, transport, adenosine triphosphate (ATP) biosynthetic processes, metabolic processes, and ATP synthesis coupled proton transport. However, although the number of up-regulated genes was larger than that of down-regulated genes, the P-value for oxidation-reduction processes was much lower than that of the immune system process (6.21E-24 *vs.* 2.49E-18; **Figure 4B**). These results suggested that the main function of the DEGs was in the pathways related to reducing the energy metabolism, although the immune system continued to be activated after three days. The CCs of these DEGs were mainly concentrated in the mitochondria (**Figure 4C**). More remarkable results were shown in the KEGG analysis, where up-regulated DEGs could hardly be effectively aggregated and were mostly concentrated in the energy metabolism pathway (**Figure 4D**).

**Figure 5:**
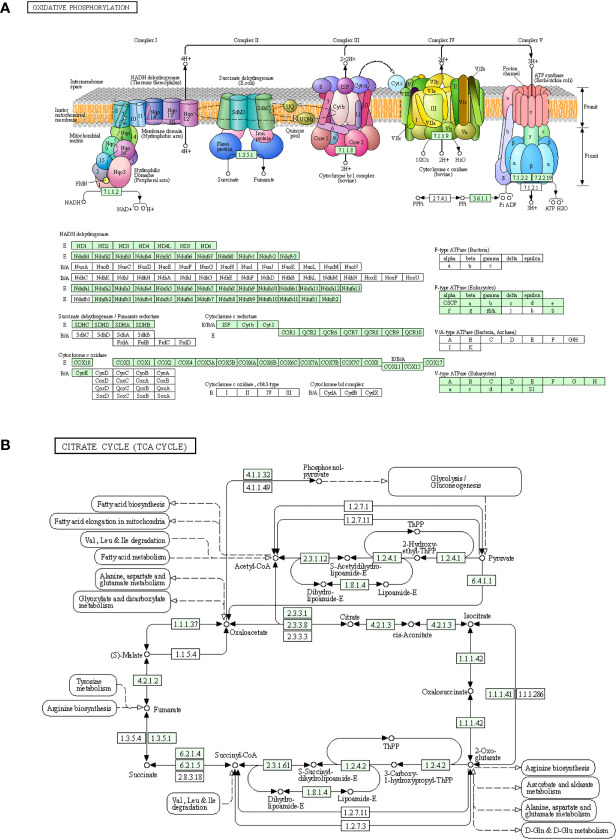
Down-regulated genes in the process of oxidative phosphorylation and the tricarboxylic acid cycle. **(A)** Genes involved in the oxidative phosphorylation pathway. **(B)** Genes involved in the citric acid cycle pathway. The newly discovered down-regulated genes after three days of interleukin-6 infusion are marked in green. ATP, adenosine triphosphate; TCA, tricarboxylic acid cycle.

Skeletal muscle is an important organ for energy supply, and the processes of oxidative phosphorylation and tricarboxylic acid cycle are the key links in the supply of ATP (32). Among the down-regulated DEGs, a large number of genes were involved in the oxidative phosphorylation pathway and exerted a strong impact on NADH dehydrogenase, succinate dehydrogenase/fumarate reductase, cytochrome C reductase, cytochrome C oxidase, and F-type ATPase (eukaryotic) (**Figure 5A**). Many down-regulated DEGs were involved in coding key enzymes in the tricarboxylic acid cycle, such as dihydrolipoamide S-succinyltransferase coding genes (Dlst, Dlat), isocitrate dehydrogenase coding genes Idh3a/b/g, and succinate dehydrogenase complex subunit coding genes Sdha/b/c/d (**Figure 5B**).

### Identification of DETFs

**Figure 6:**
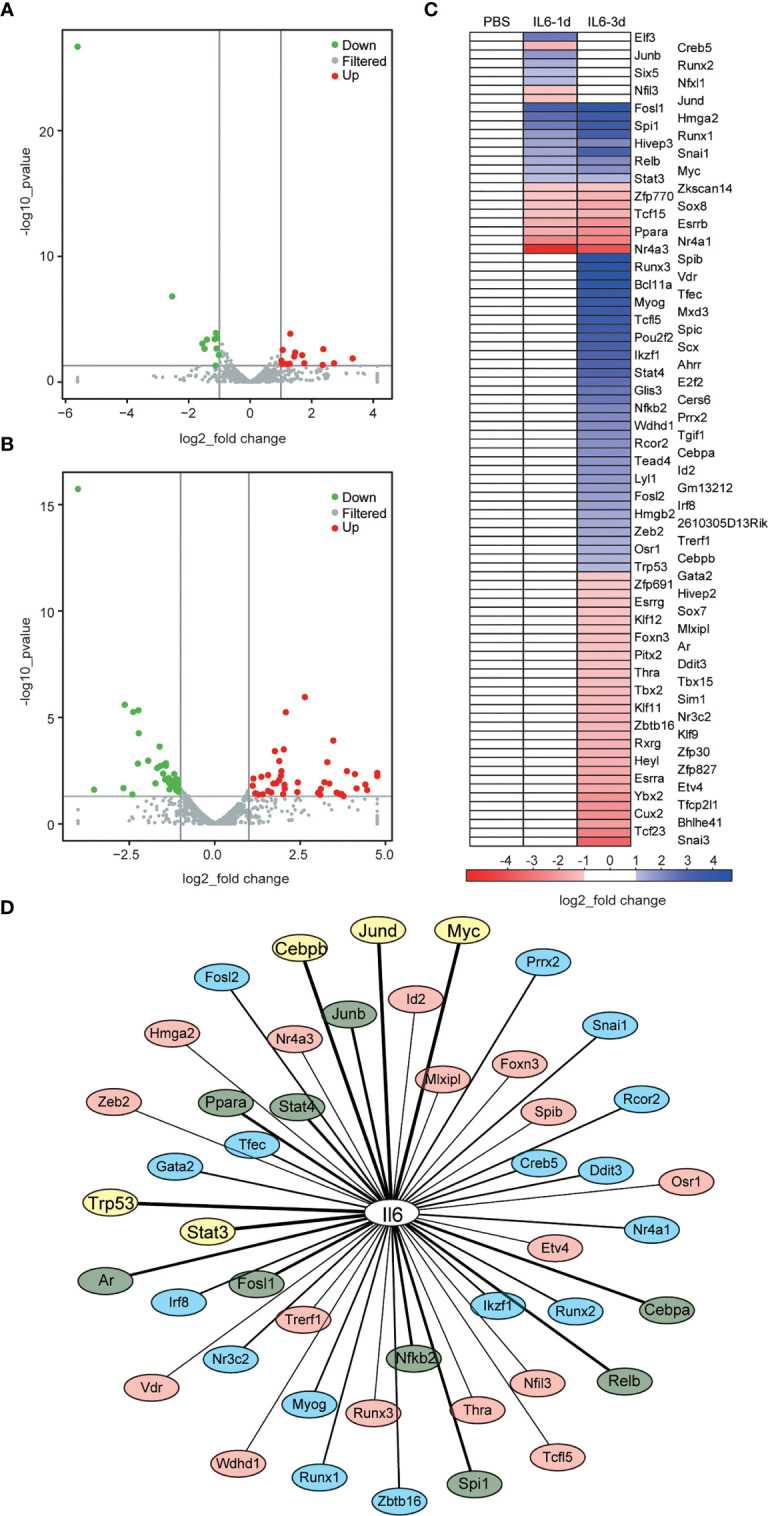
Identification of differentially expressed transcription factors (DETFs) and interaction between interleukin-6 (IL-6) and transcription factors (TFs). **(A, B)** Volcano graphs representing the DETFs after one day **(A)** and three days **(B)** of IL-6 infusion. Red indicates up-regulated expression, and green indicates down-regulated expression. **(C)** Heat map of the DETFs, which are classified according to their differential expression after one day of IL-6 infusion, their co-differential expression after one and three days of IL-6 infusion, and their differential expression after three days of IL-6 infusion. Blue indicates up-regulated expression, and green indicates down-regulated expression. **(D)** Interaction analysis between IL-6 and DETFs. Interaction between IL-6 and DETFs is ranked from strong to weak: TFs with a score > 800 are marked in yellow, 800 > score > 500 marked in green, 500 > score > 300 marked in blue, and a score < 300 marked in light red. down, down-regulated; up, up-regulated; IL-6, interleukin-6; PBS, phosphate-buffered saline; TF, transcription factor.

The DEGs did not show a direct connection with IL-6, although its infusion caused immune receptor activation and reduced energy metabolism. As a driver of gene expression, transcription factors may play an important role in these processes. There were 14 up-regulated DETFs and 11 down-regulated DETFs after one day of IL-6 infusion, and 45 up-regulated DETFs and 39 down-regulated DETFs after three days (**Figures 6A, B**). We used heatmaps to analyze the dynamic changes of these DETFs after one and three days of IL-6 infusion, consistent with the classification of DEGs. There were only 8 distinct DETFs after one day of IL-6 infusion, 17 DETFs were co-expressed after one and three days, and 67 further DETFs occurred after three days only (**Figure 6C**). Among them, several DETFs that were closely related to skeletal muscle atrophy, such as Stat3 and Myog, were up-regulated.

The Cytoscape analysis showed that 46 (50%) of the 92 identified DETFs had potential interactions with IL-6, among which Stat3, Trp53, Myc, Junb, Spi1, Nfkb2, Stat4, Relb, Jund, Ppara, Rcor2, Irf8, Runx1, Runx2, and Tfec had strong effects on IL-6 (**Figure 6D** and **Supplementary File 2**).

### Interaction Between DETFs and DEGs

**Figure 7:**
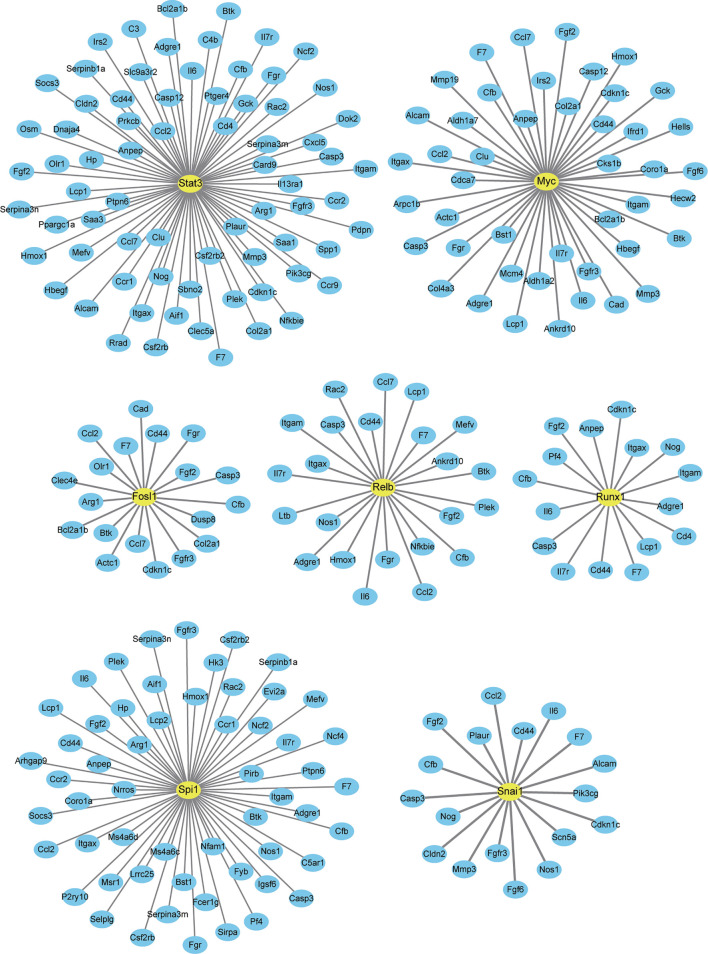
Interaction between differentially expressed genes (DEGs) and differentially expressed transcription factors co-expressed after one and three days of interleukin-6 (IL-6) infusion. All transcription factors (TFs) and DEGs co-differentially expressed after one and three days of IL-6 infusion are integrated and analyzed. Several representative TFs that interacted strongly with IL-6 are labeled. Only TFs and DEGs with a score > 200 for the interaction are shown.

**Figure 8:**
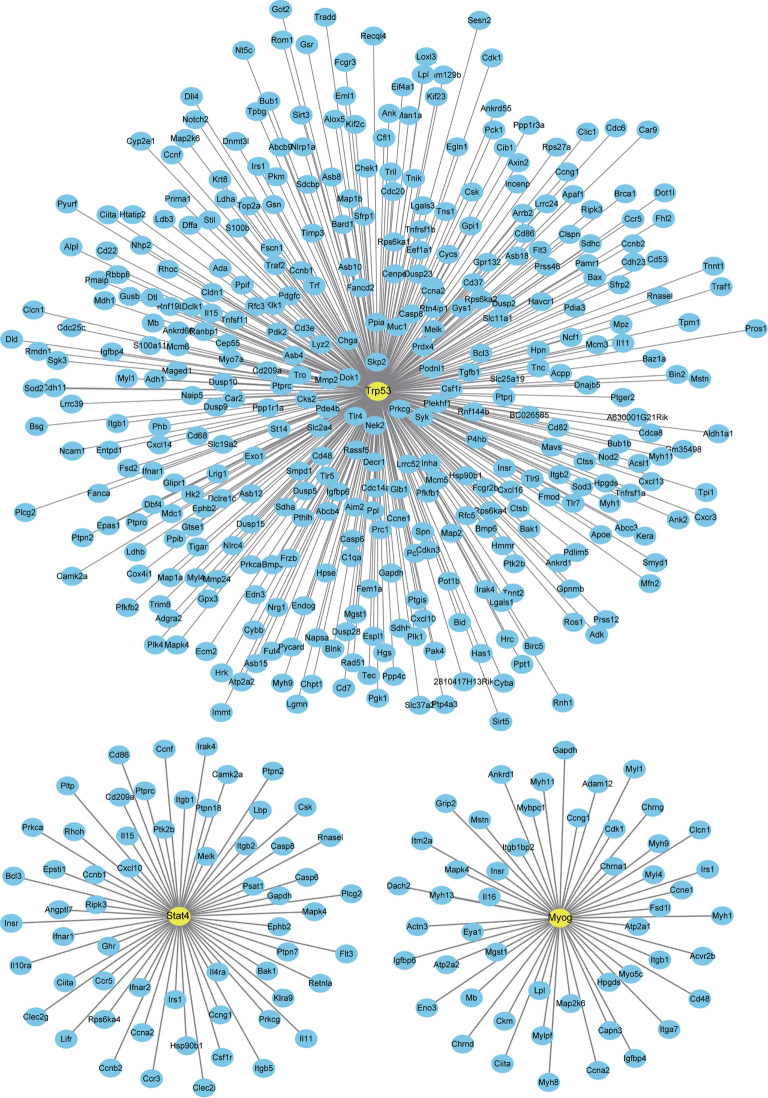
Interaction between newly discovered differentially expressed genes (DEGs) and differentially expressed transcription factors (DETFs) after three days of interleukin-6 (IL-6) infusion. All the newly discovered DEGs and DETFs after three days of IL-6 infusion are integrated and analyzed. The three representative transcription factors (TFs) marked can interact strongly with IL-6 or have been reported to be related to skeletal muscle atrophy. Only TFs and DEGs with a score > 200 for the interaction are shown.

Finally, we analyzed a potential interaction between DETFs and DEGs co-expressed after one and three days of IL-6 infusion and between those that were newly discovered after three days of IL-6 infusion. There were 660 potential interactions between the 17 DETFs and 359 DEGs found after one and three days of IL-6 infusion. Representative DETF-mediated interactions are shown in **Figure 7** and the **Supplementary File 3**. For the 67 DETFs and 1748 DEGs that were newly discovered after three days of IL-6 infusion, a total of 6192 potential interactions was found (**Supplementary File 4**). The DETF Trp53 with the most complex interaction relationship and the other two representative ones, Stat4 and Myog, are shown separately in **Figure 8**.

## Discussion

Inflammation is involved in a variety of physiological processes. Uncontrolled and delayed inflammation is a common feature of many metabolic diseases, including skeletal muscle and neurological diseases (33, 34). A study conducted in patients with non-small cell lung cancer (NSCLC) and muscle atrophy pointed out that the overexpression of IL-6 and IL-8 can be potentially used as biomarkers of a poor prognosis in patients with NSCLC (35). Anti-inflammatory drugs, including aspirin, pyrroloquinoline quinone, and isoquercitrin, can alleviate skeletal muscle atrophy (36–38). In the Lewis lung cancer mouse model, luteolin inhibits the expression of genes related to muscle breakdown by alleviating inflammation, thereby protecting the loss of skeletal muscle caused by cachexia (39).

In this study, we established a mouse model of skeletal muscle atrophy induced by continuous infusion of IL-6 and analyzed early gene expression changes using transcriptome sequencing technology. We found two mechanisms by which IL-6 causes skeletal muscle atrophy: by activating multiple immune receptors and inhibiting energy metabolism. Furthermore, we analyzed the causes of these two mechanisms and found multiple potential transcription factor targets downstream of IL-6.

In addition to maintaining the body’s athletic ability, skeletal muscle is also considered to be an endocrine organ that can release a variety of cytokines (such as IL-6) (40). These effects of IL-6 for skeletal muscle are a double-edged sword. On the one hand, excessive IL-6 can cause skeletal muscle atrophy (18). On the other hand, the level of IL-6 in peripheral blood may increase by nearly 100 times after exercise, which stimulates muscle building without signs of muscle damage (41). IL-6 is considered to activate muscle satellite cells and initiate myocyte regeneration (42), likely depending on the level of IL-6 in muscle tissue. The findings from this study deepen our understanding of IL-6’s beneficial and harmful effects on muscle tissue and identify its role as a factor promoting muscle atrophy.

We focused on the changes in skeletal muscle during the early stages of IL-6 infusion because of the quick onset of IL-6 action and function. IL-6 exerts its functions through classical signaling and trans-signaling. Classic signaling is initiated by IL-6 binding to the membrane-bound IL-6 receptor (IL-6R; CD126), followed by subsequent binding to the homodimer of gp130 and transmission of intracellular signals. Although gp130 is widely expressed, IL-6R is only expressed on hepatocytes, neutrophils, monocytes/macrophages, and some lymphocytes (12). However, alternative splicing causes the deletion of transmembrane domains, and many membrane receptors, including IL-6R, therefore have soluble subtypes that are secreted into body fluids (43). Soluble IL-6R cannot only bind to IL-6 but also to the gp130 homodimer, inducing IL-6 trans-signaling (12). These observations provide the theoretical base of IL-6’s direct action on skeletal muscle cells and subsequent effects. It is worth noting that due to the tissue-wide combination of IL-6 and IL-6R, such a massive infusion of IL-6 may cause off target effects in other tissues. However, skeletal muscle, as a direct injected target, receives the most IL-6, so the minor effects of this potential bias will not interfere with the key conclusions of this paper. Increasing IL-6R may aggravate the effect of IL-6. Our previous studies have confirmed that the antibody against IL6R can alleviate denervated muscle atrophy (20). Therefore, reducing the content of IL-6R in skeletal muscle may also alleviate the skeletal muscle atrophy after IL-6 injection, we will pay attention to this potential treatment strategy in the next work.

The continuous up-regulation of IL-6 mRNA after one and three days of IL-6 infusion suggests that exogenous IL-6 stimulated the secretion of endogenous IL-6. Studies have shown that the injection of recombinant IL-6 into cultured mouse and human myotubes can increase the muscle IL-6 mRNA expression (44, 45). These *in vitro* findings are consistent with the results of our *in vivo* experiments in this study. Interestingly, we found a large number of other up-regulated immune receptor-encoding genes on the muscle cells’ membrane. Nfkb1, Nfkb2, RelA, and RelB are the coding genes of the main subunits of the nuclear factor κ-light-chain-enhancer of activated B cells (NF-κB). Subsequent analysis showed that Nfkb1, Nfkb2, RelA, and RelB were all up-regulated and considered as downstream transcription factors that interacted strongly with IL-6 (Nfkb1 and RelA were up-regulated 1.43 and 1.34 fold, which is not shown in the figure). NF-κB is a classic activator of inflammation, controlling the release of many inflammatory factors such as IL-1, IL-6, and TNFα. The luciferase reporter assay of the IL-6 promoter has shown that IL-6 expression (or stimulation) through the autocrine pathway depends on the activation of NF-κB (46). Furthermore, NF-κB is considered to be a key factor in muscle atrophy in that NF-κB signaling regulates the release of cytokines and chemokines from skeletal muscle cells (47). NF-κB activation is also considered to be a key factor in initiating protein degradation and modulating muscle atrophy (48). In summary, IL-6 injection may activate transcription factors, such as NF-κB. Through positive feedback loops, substantial amounts of inflammatory factors are released, and receptors of these inflammatory factors are activated. The continued inflammatory response eventually triggers skeletal muscle atrophy. It is worth noting that the membrane receptor Toll-like receptor 4 (TLR4) has been reported to mediate cancer-induced muscle atrophy by activating the protein degradation pathway in the muscle of Lewis lung cancer (LLC) mice and stimulating the innate immune response (49).

IL-6 is known to cause skeletal muscle atrophy. Reduced energy metabolism is also known to play a role in skeletal muscle atrophy (especially disuse atrophy). In this study, we, for the first time, demonstrated that IL-6 promotes skeletal muscle atrophy by reducing energy metabolism. IL-6 infusion for three days downregulated the expression of multiple genes located in the mitochondria, and the two main energy metabolism pathways, oxidative phosphorylation and the citric acid cycle, were inhibited.

Similar phenomena were found in overweight elderly people with sarcopenia who had insufficient production of muscular ATP and deficient glycolysis and oxidative phosphorylation metabolic pathways (50). Studies have shown that reduced ATP production will increase adenosine 5’-monophosphate (AMP), and upregulated intracellular AMP levels lead to the activation of AMP-activated protein kinase (AMPK) (51). AMPK can phosphorylate FoxO3 at the Ser588 site to promote the expression of atrophic genes involved in the ubiquitin-proteasome and autophagy-lysosome systems, thereby facilitating muscle atrophy (52, 53). Additionally, mitochondria-targeted antioxidant drugs have been shown to eliminate reactive oxygen species and significantly alleviate the atrophy phenotype in denervated and inactivity-induced skeletal muscle atrophy models (54, 55). The findings of the present study indicate that mitochondrial dysfunction and accumulation of reactive oxygen species may occur in the inflammation downstream.

Multiple transcription factors interact with IL-6 and have the potential to regulate the expression of DEGs. These factors and downstream molecules of IL-6 may become new targets for the therapy of muscle atrophy. Among them, the JAK/STAT pathway has been confirmed to interact with IL-6 in a variety of muscle atrophy models. Madaro et al. (31) reported that denervation leads to the progressive accumulation of fibro-adipogenic progenitor cells, continuous STAT3 activation, and the secretion of high levels of IL-6, thereby promoting muscle atrophy and fibrosis. We previously found that high levels of IL-6 can exacerbate C2C12 myotube atrophy by activating the JAK/STAT3 pathway, while ruxolitinib, a JAK1/2 inhibitor, and C188-9, a STAT3 inhibitor, can significantly reduce IL-6 induced C2C12 myotube atrophy by inhibiting the JAK/STAT3 pathway (20).

The myogenin (MyoG) transcription factor was considerably up-regulated after three days of IL-6 infusion and predicted to interact with IL-6 directly. Interestingly, Myog is a marker of muscle satellite cells related to cell differentiation and extremely important in embryonic development. The deletion of MyoG is lethal for the embryo (56, 57). The up-regulation of MyoG expression indicates a proliferation of muscle satellite cells. However, studies have shown that MyoG is also up-regulated in the denervated muscle atrophy model and regulates the expression of E3 ubiquitin ligase MuRF1 and atrogin-1, which promote muscle proteolysis and atrophy (58). In this study, the expression of MyoG had a similar pattern to that seen in denervation, which may further support the conclusion that IL-6 acts as an early trigger of skeletal muscle atrophy.

**Figure 9:**
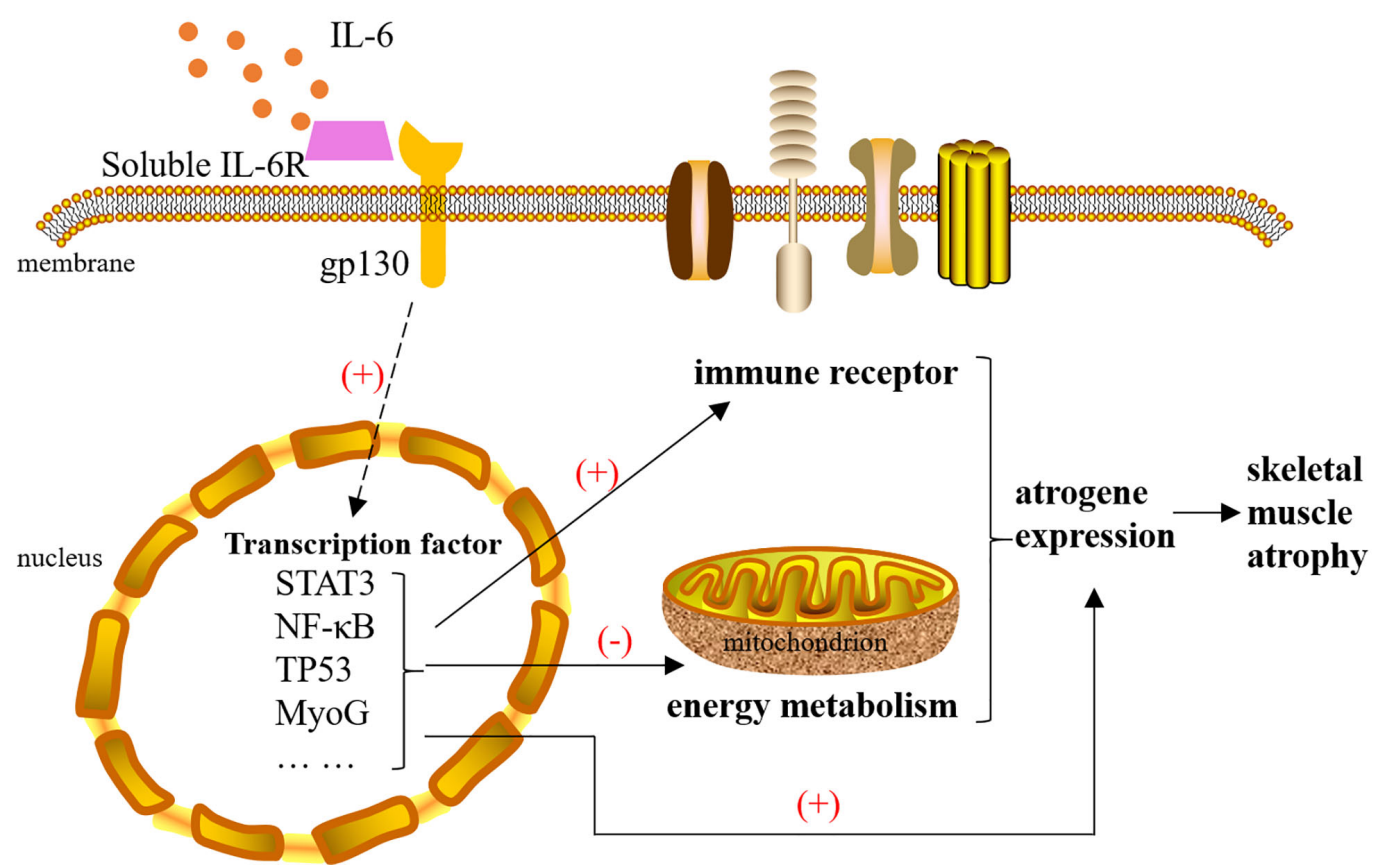
The mechanism of interleukin-6 (IL-6) induced skeletal muscle atrophy. IL-6 signals are transmitted to the nucleus with the help of IL-6R and gp130, causing the activation of transcription factors, such as STAT3, NF-κB, TP53, and MyoG. These transcription factors cause changes in the expression of atrophy genes in three ways: 1) They can directly act on atrogenes. 2) They activate multiple immune receptors located on the cell membrane to amplify the immune response. 3) They inhibit mitochondrial function by reducing the expression of energy metabolism genes. Changes in the expression of atrogenes will cause skeletal muscle atrophy. IL-6, interleukin-6.

This study illustrates the two main molecular mechanisms underlying IL-6-induced skeletal muscle atrophy that were immune receptor activation and a reduction of the energy metabolism (**Figure 9**). Our next studies will focus on the role and mechanisms of IL-6 downstream transcription factors in the occurrence of muscle atrophy, endeavoring to discover new targets of muscle atrophy treatment beyond suppressing inflammation and enhancing mitochondrial function.

## Data Availability Statement

The data presented in the study are deposited in the ArrayExpress repository, accession number E-MTAB-10794.

## Ethics Statement

The animal study was reviewed and approved by a project license (No. S20200312-003) granted by the Animal Ethics Board of Nantong University.

## Author Contributions

Conception and design: LL and XG. Administrative support: LL and XG. Provision of study materials or patients: HS, JS, ML, LQ, LZ, ZH, YS, and BL. Collection and assembly of data: HS, JS, LZ, ZH, YS, and BL. Data analysis and interpretation: HS, JS, ZH, YS, and BL. All authors contributed to the article and approved the submitted version.

## Funding

This work was supported by the National Natural Science Foundation of China (Nos. 82072160, 31730031, 32130060, 92068112, 32000841 and 81901933), the National Key Research and Development Program of China (No. 2017YFA0104703), the Major Natural Science Research Projects in Universities of Jiangsu Province (No. 20KJA310012), the Natural Science Foundation of Jiangsu Province (Nos. BK20202013, BK20201209), the “QingLan Project” in Jiangsu Universities, the Priority Academic Program Development of Jiangsu Higher Education Institutions.

## Conflict of Interest

The authors declare that the research was conducted in the absence of any commercial or financial relationships that could be construed as a potential conflict of interest.

## Publisher’s Note

All claims expressed in this article are solely those of the authors and do not necessarily represent those of their affiliated organizations, or those of the publisher, the editors and the reviewers. Any product that may be evaluated in this article, or claim that may be made by its manufacturer, is not guaranteed or endorsed by the publisher.
